# Orthodontic treatment of an adult Class II division 1 malocclusion with nonextraction assisted by lip myofunctional training: A case report

**DOI:** 10.1002/ccr3.2859

**Published:** 2020-04-14

**Authors:** Jianhua Hou, Xiuping Meng

**Affiliations:** ^1^ Department of Orthodontics Hospital of Stomatology Jilin University Changchun China; ^2^ Department of Endodontics Hospital of Stomatology Jilin University Changchun China

**Keywords:** adult treatment, Class II malocclusion, esthetics, myofunctional therapy

## Abstract

Consideration of facial soft tissue is critical in orthodontic diagnosis and treatment planning to achieve improvement in facial esthetics as well as dental occlusion. As is illustrated by success in orthodontic treatment of an adult Class II division 1 malocclusion with nonextraction and assisted by lip myofunctional training.

## Graphical abstract

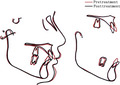

## 1 INTRODUCTION

Facial esthetics is an important goal in orthodontic treatment.^1^, ^2^ It should be recognized that success in orthodontic treatment depends much on improvement of facial soft tissues as well as skeletal and dental tissues.^2^ The patients with Class II division 1 malocclusion often have problems in both dental tissue and facial soft tissues, typically the protrusion of upper incisors and lip, which could have impact on facial esthetics.^2^ It has been reported that perioral soft‐tissue profile, especially the lip form, could be changed by anterior teeth retraction after orthodontic treatment.^3^, ^4^ However, the correlation between lip change and incisor retraction could be influenced by some factors such as growth, treatment modalities, individual soft‐tissue traits, and oral habits.^2^, ^4^, ^5^, ^6^, ^7^, ^8^, ^9^ For instance, changes of facial profile in patients treated with nonextraction could be different from those with extraction.^5^, ^6^ The response of lip to incisor retraction could vary among patients with different lip thickness.^2^, ^7^, ^8^ Moreover, in patients with lip incompetence resulting from lip‐biting habit, perioral soft tissues could hardly be changed solely by orthodontic treatment.^9^ In such cases, orthodontic treatment should be assisted by myofunctional training, which has been reported to be effective during the treatment of myofunctional disorders.^9^, ^10^, ^11^, ^12^ Therefore, it is critical to evaluate characteristics of soft tissues as well as skeletal and dental tissues in orthodontic treatment planning.^5^, ^7^ This report is to present the nonextraction treatment of an adult Class II division 1 malocclusion combined with lip myofunctional training to meet the patient's esthetic and functional expectations.

## 2 METHODS AND MATERIALS

### 2.1 Clinical examination

**FIGURE 1:**
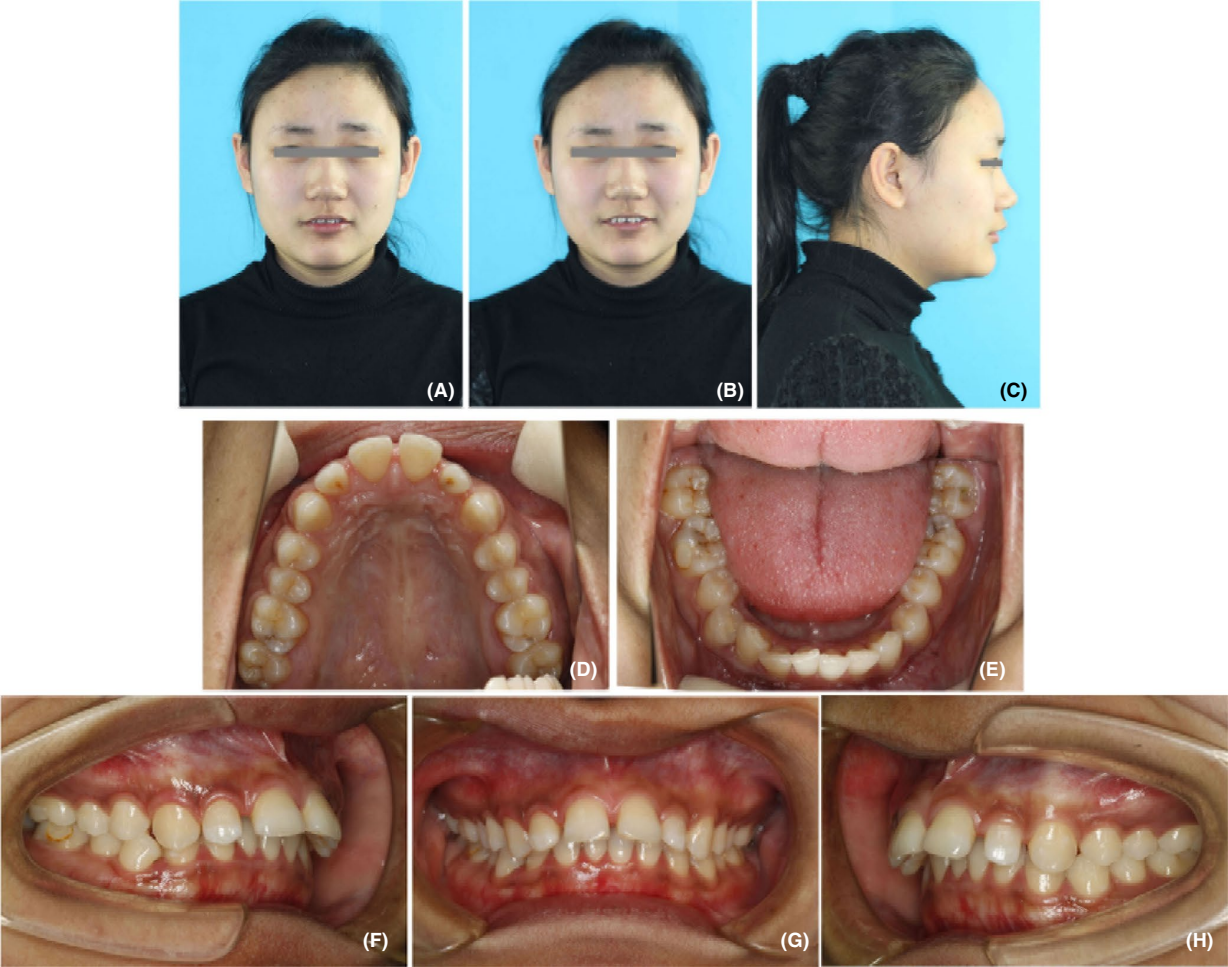
Pretreatment photographs. A‐C, facial photographs; D‐H, intraoral photographs

The patient was a 22‐year‐old Chinese female with chief complaints of protrusive upper teeth and incompetent lip. Her medical history was unremarkable. She had a habit of lower lip biting. The extraoral examination exhibited an acceptable profile convexity with a slightly retrusive mandible, a lack of passive lip seal, protrusive upper lip, acute nasolabial angle, and deep labiomental fold (Figure 1A‐C). There were no temporomandibular joint symptoms, and mandibular movement was normal.

**FIGURE 2:**
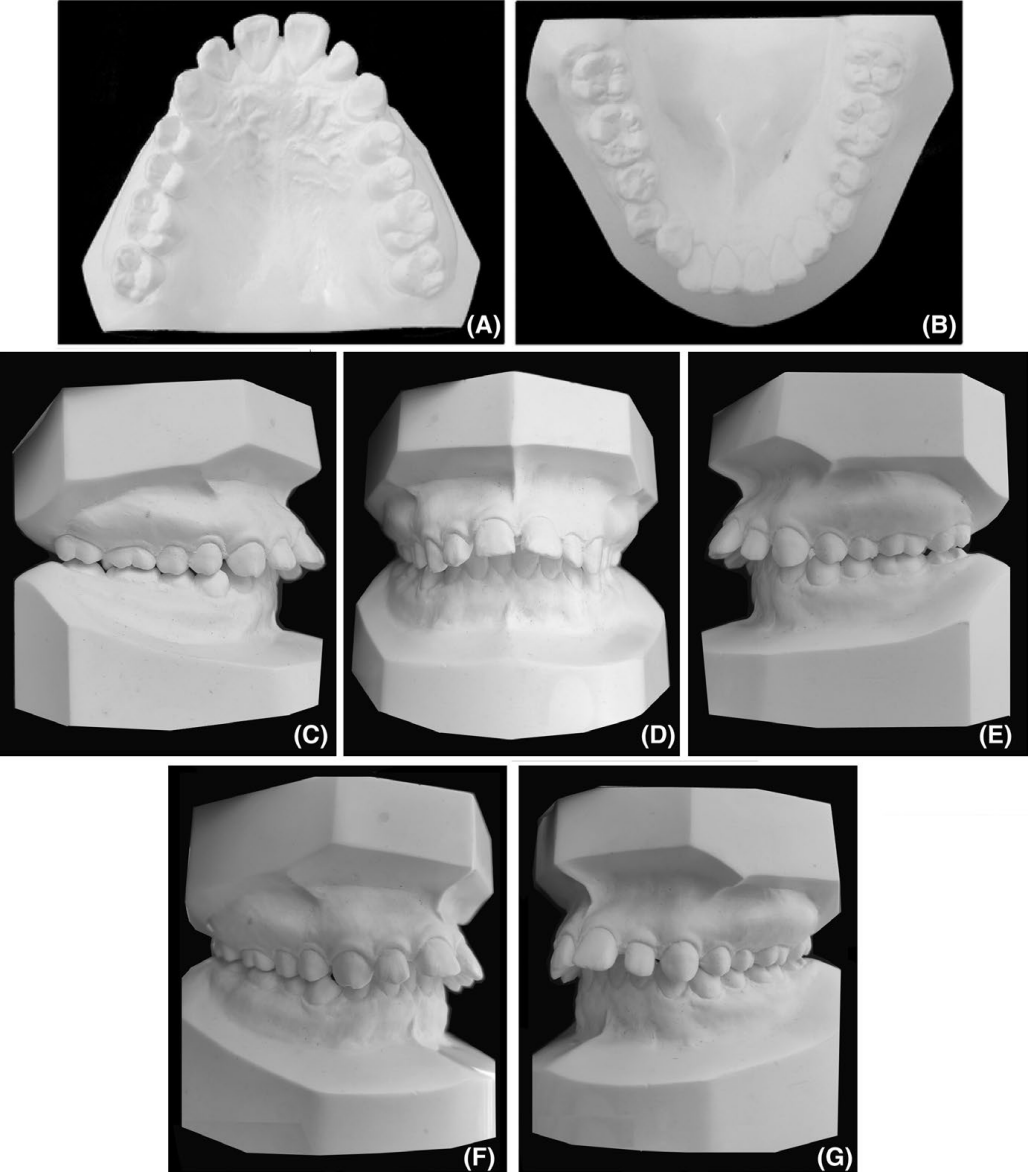
Pretreatment dental casts. C and E, lateral occlusion viewing perpendicular to the buccal surface of molars; F and G, lateral occlusion viewing at almost the same angle as in Figure 1F and H

Dental casts revealed Class II molar and canine relationships (Figure 2C and E). She had a 10‐mm overjet and a 4‐mm overbite (Figures 1 and 2). The dental midline coincided with the facial midline. The upper arch was tapered in shape with anterior diastemata. The lower arch was constricted with 4mm of crowding. The curve of Spee was deep with the depth of 5 mm. Most teeth were worn to different extent. Model analysis indicated the discrepancy of Bolton index which was 81.7% in anterior teeth and 94% in total teeth.

**FIGURE 3:**
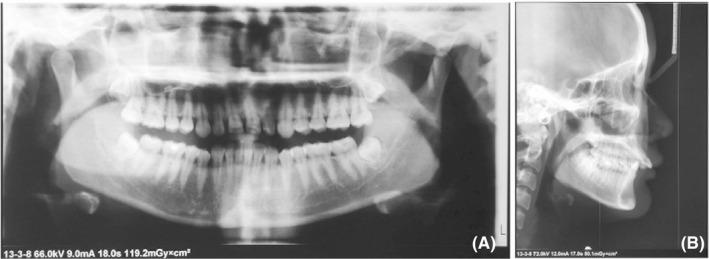
Pretreatment panoramic and lateral cephalometric radiographs

The panoramic radiograph showed the presence of third molars which were under development (Figure 3A). The cephalometric analysis showed an acceptable profile convexity with a slightly retrusive mandible (SNB = 76.5°), significant upper incisor proclination (U1/NA = 42.5°), and lower incisor lingual inclination (L1/NB = 15.0°). The basic soft‐tissue chin position was excellent. The upper lip was protrusive (H angle = 18.0°, nasolabial angle = 82.0°, superior sulcus depth = 4.5 mm, subnasale to H line = 10.0 mm). The upper lip strain was insufficient (upper lip strain = 0.0 mm) because the upper lip thickness was relatively excessive compared with the basic lip thickness. The lower lip was retrusive (lower lip to H line=−2.5 mm, inferior sulcus depth = 10.0 mm) with its greater thickness (lower lip thickness = 16.5 mm). The soft‐tissue chin was slightly thicker (soft‐tissue chin thickness = 13.5 mm) (Figure 3B; Table 1). The patient was diagnosed as a Class II division 1 malocclusion with poor esthetics in perioral soft tissues.

**TABLE 1:** Cephalometric measurements

Measurements	Norms	Pretreatment	Posttreatment
Skeletal measurements			
SNA (°)	82.8 ± 4.0	80.5	79.5
SNB(°)	80.1 ± 3.9	76.5	75.5
ANB(°)	2.7 ± 2.0	4.0	4.0
MP/SN (°)	32.5 ± 5.2	29.5	30.0
FMA (°)	31.1 ± 5.6	21.0	21.0
A‐NP (mm)	−3.0 ~ 5.0	2.5	3.0
Dental measurements			
U1/NA (°)	22.8 ± 5.7	42.5	13.0
U1/SN (°)	105.7 ± 6.3	122.5	93.0
L1/NB (°)	30.5 ± 5.8	15.0	36.0
IMPA (°)	93.9 ± 6.2	89.0	109.0
U1/L1 (°)	124.2 ± 8.2	119.5	126.5
Soft‐tissue measurements			
Soft‐tissue facial angle (°)	91.0 ± 7.0	90.0	90.0
Nasolabial angle (°)	90.0 ± 12.0	82.0	95.0
H angle (°)	11.0 ± 4.13	18.0	16.5
Nose prominence (mm)	19.0 ± 5.0	7.5	10.5
Superior sulcus depth (mm)	1.0 ~ 4.0	4.5	3.0
Subnasale to H line (mm)	5.0 ± 2.0	10.0	8.0
Basic upper lip thickness (mm)		12.0	11.5
Upper lip thickness (mm)		12.0	12.0
Upper lip strain (mm)	1.0 ± 0.5	0.0	−0.5
Lower lip thickness (mm)		16.5	12.0
Lower lip to H line (mm)	0.5 ± 1.5	−2.5	−1.5
Inferior sulcus to H line (mm)	5.0 ± 1.0	10.0	7.5
Soft‐tissue chin thickness (mm)	11.0 ± 1.0	13.5	12.0
Upper lip to E line (mm)	−1.3 ± 2.0	0.5	−1.5
Lower lip to E line (mm)	−2.0 ± 2.0	−2.8	−1.8

Note

Soft‐tissue facial angle, angle between FH plane and the line drawn from soft‐tissue Na (Na’) to the soft‐tissue chin at a point overlying the hard‐tissue suprapogonion of Ricketts; nasolabial angle, angle between the line representing the lower border of nose and the one representing the inclination of upper lip; H angle, angle between H line and Na’ soft‐tissue pogonion (Pog’) line; nose prominence, distance from nose tip to the line perpendicular to FH and running tangent to the vermilion border of upper lip; superior sulcus depth, distance from the deepest point of the incurvation of superior sulcus to H line; basic upper lip thickness, the upper lip thickness at the level about 3 mm below point A; upper lip thickness, the upper lip thickness overlying incisor crowns at the level of vermilion border; upper lip strain, the difference between basic upper lip thickness and upper lip thickness; lower lip thickness, distance from labrale inferius (Li) to the most prominent labial point of L1; lower lip to H line, distance from lower lip to H line; inferior sulcus to H line, distance from the deepest point of the incurvation between the vermilion border of lower lip and soft‐tissue chin to H line; upper lip to E line, distance from the upper lip to the line connecting the tip of nose and Pog’; lower lip to E line, distance from the lower lip to E line.

Abbreviations: ANB, A point‐Na‐B point; FMA, angle between Frankfort horizontal (FH) plane and MP;IMPA, angle between L1 axis and MP; L1/NB, angle between the lower central incisor (L1) axis and Na‐B line; MP/SN, angle between mandibular plane (MP) and S‐Na line; ‐NP, distance from A point to Na‐Pogonion (Pog) line; SNA, sella (S)‐nasion (Na)‐A point; SNB, S‐Na‐B point; U1/L1, angle between U1 and L1 axis; U1/NA, angle between the upper central incisor (U1) axis and Na‐A line; U1/SN, angle between U1 axis and S‐Na line.

### 2.2 Treatment objectives

The treatment objectives for this patient were to (a) improve the facial esthetics, especially in lip tissues, (b) retract the upper incisors and achieve ideal overjet, (c) level the Spee curve and establish proper overbite, (d) normalize the shape of both arches and make them coordinate with each other, and (e) close the diastemata in upper arch and relieve the crowding of lower arch.

### 2.3 Treatment plan

The plan was orthodontic treatment which was nonextraction assisted by lip myofunctional training.

### 2.4 Treatment progress

**FIGURE 4:**
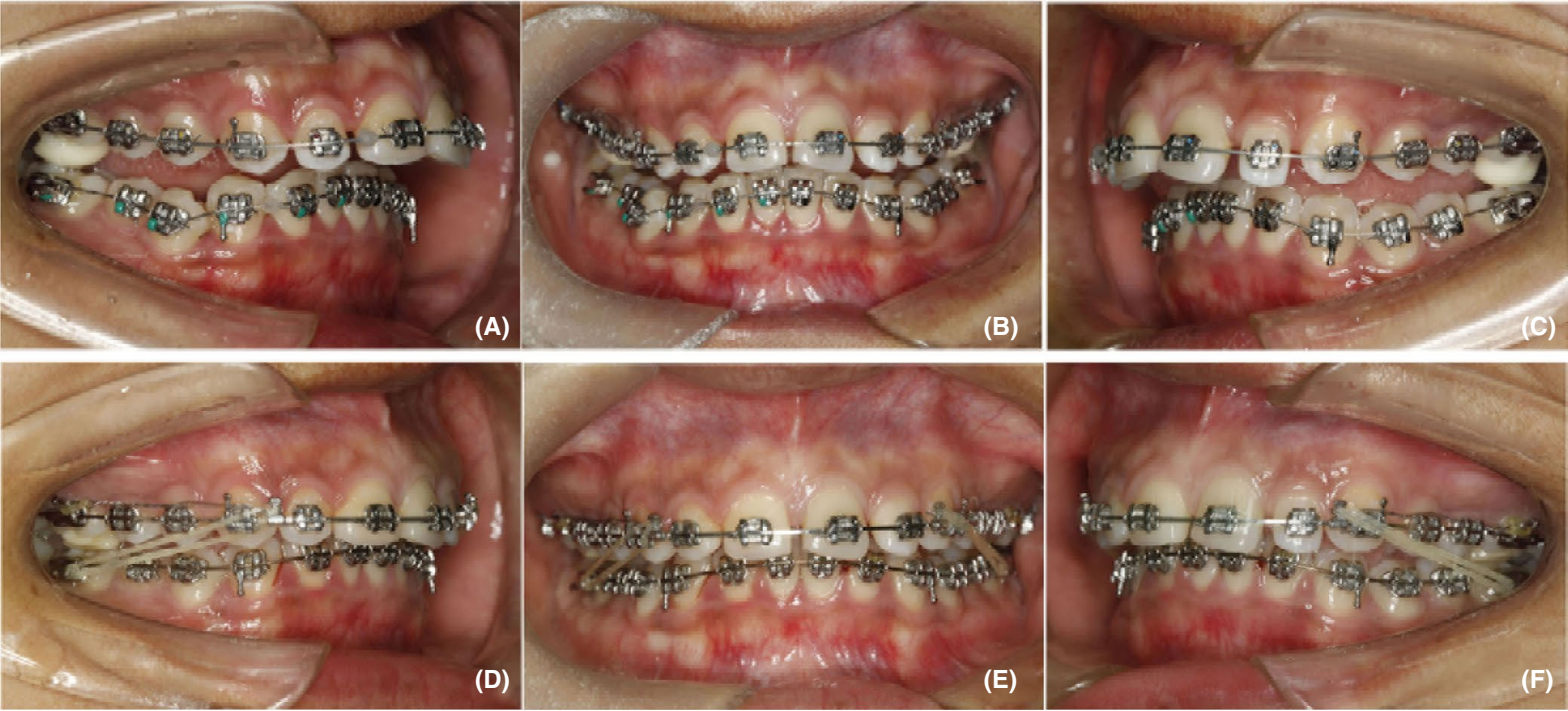
Intraoral progress photographs. A‐C, leveling of the arches and eliminating occlusal interruption by using resin raisers; D‐F, adjusting occlusal relationship with Class II elastics

At the beginning of the treatment, the patient received instructions about lip myofunctional training by using a coin, which was a practicable method and commonly used in clinical practice. The patient was instructed to close the lips and maintain a one‐yuan coin (25 mm in diameter and 6 g in weight) horizontally between upper and lower lip for at least 2 hours per day. This training was kept on all through the orthodontic treatment. Preadjusted fixed appliances (0.022 × 0.028‐inch, MBT system; 3M Unitek) were bonded, and simultaneously resin raisers were placed on the occlusal surfaces of the upper first molars to eliminate any occlusal interruption (Figure 4A‐C). Leveling and expansion were performed sequentially with 0.012‐inch nickel titanium (NiTi), 0.016‐inch NiTi, 0.016‐inch stainless steel, 0.018 × 0.025‐inch NiTi, and 0.018 × 0.025‐inch stainless steel archwires. When the archwire progressed to 0.016‐inch stainless steel archwires, Class II elastics were used from the lower first molars to upper anterior teeth to adjust the inter‐arch relationship sagittally and vertically (Figure 4D‐F). During the leveling, the height of resin raisers on upper molars was gradually reduced. After 18 months of active treatment, the appliances were removed and Hawleys retainers were used for retention.

## 3 RESULTS

**FIGURE 5:**
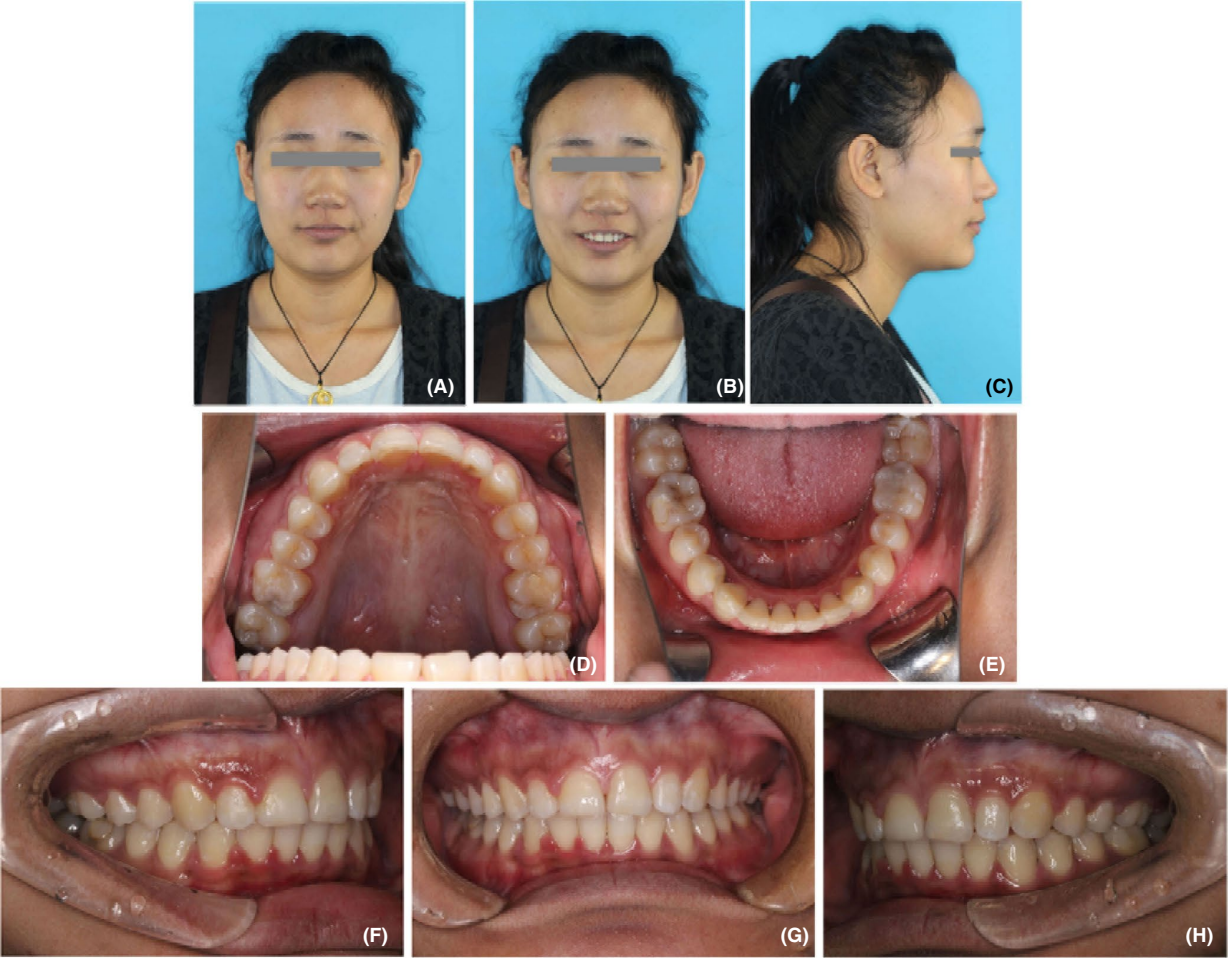
Posttreatment photographs. A‐C, facial photographs; D‐H, intraoral photographs

**FIGURE 6:**
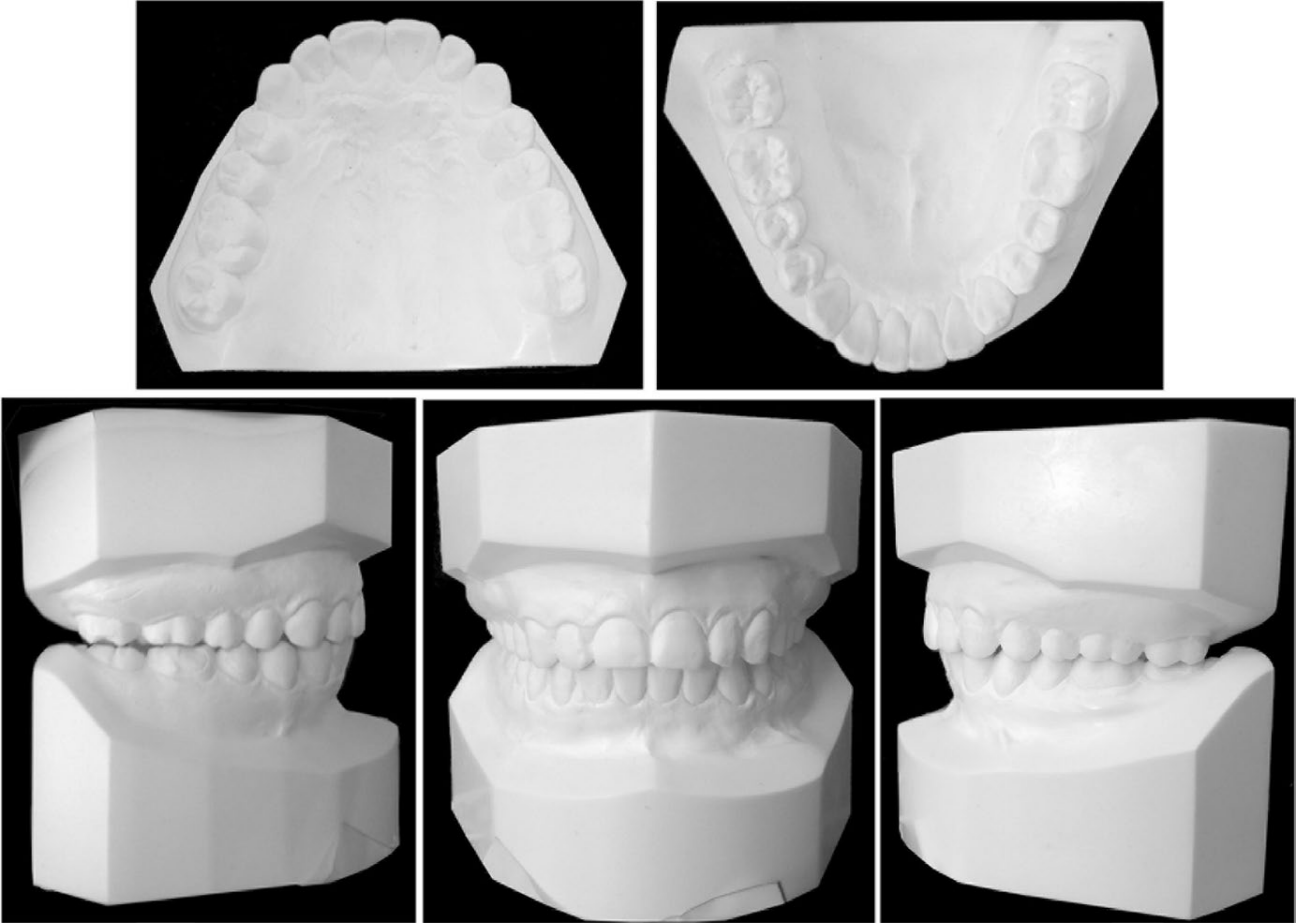
Posttreatment dental casts

**FIGURE 7:**
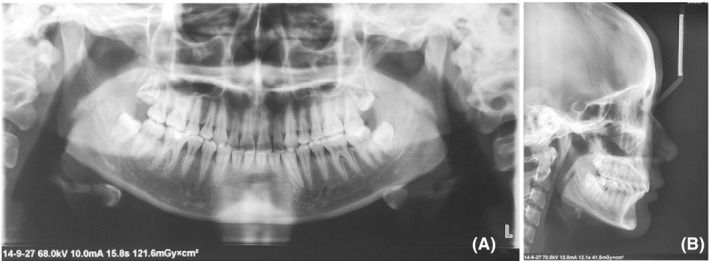
Posttreatment panoramic and lateral cephalometric radiographs

**FIGURE 8:**
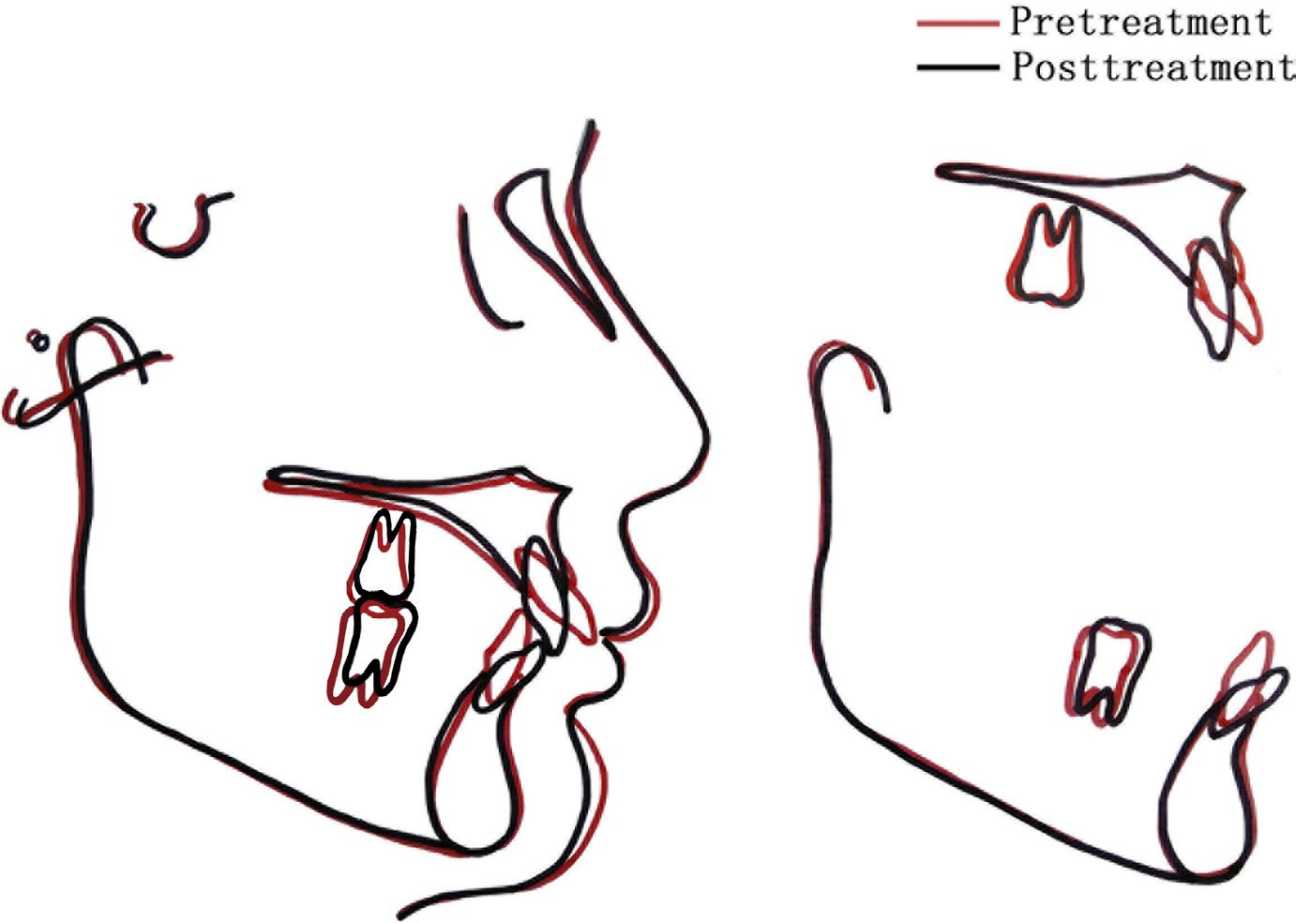
Overall superimposition of the pretreatment (red line) and posttreatment (black line) tracings

**FIGURE 9:**
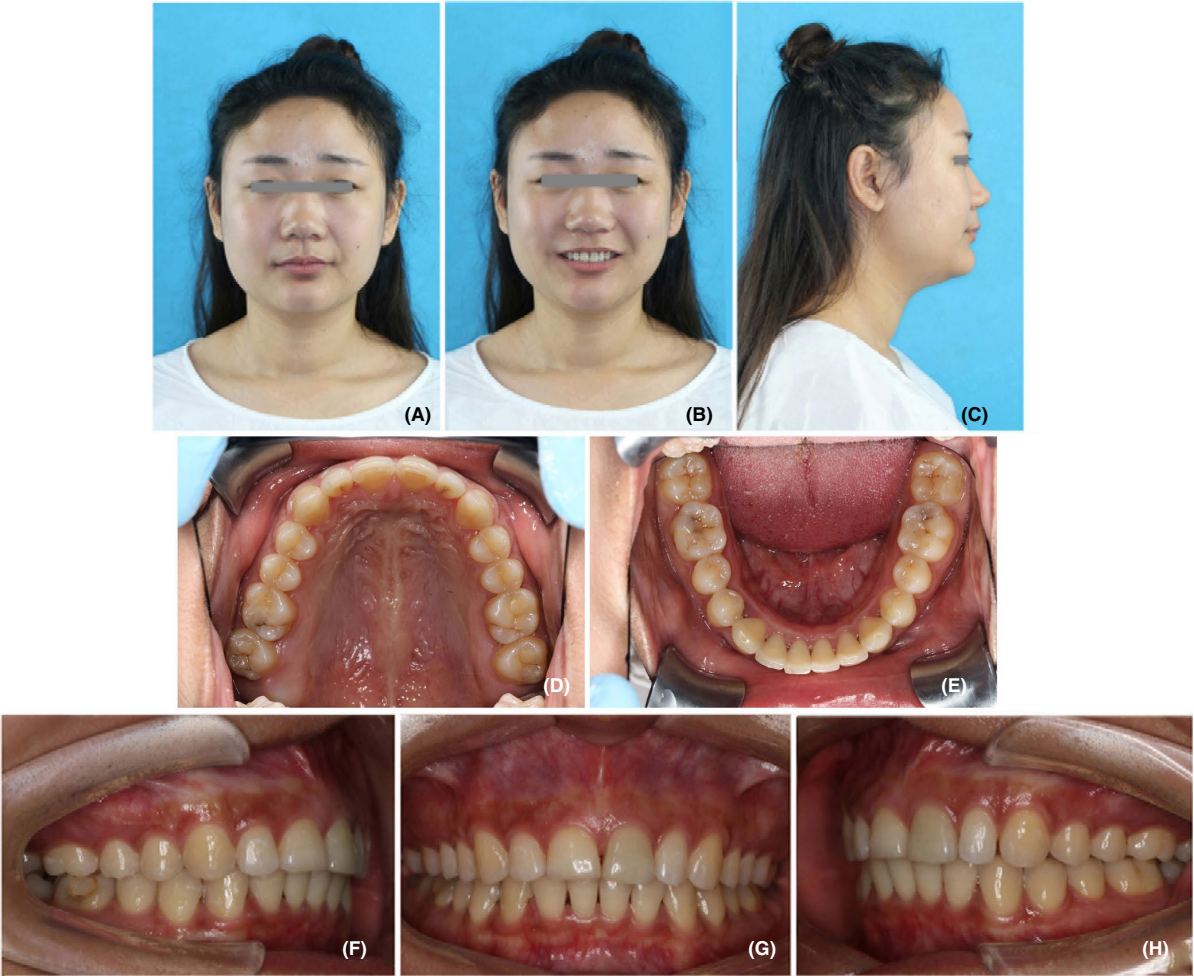
Facial and intraoral photographs at the 4‐y follow‐up

The patient's habit of lip biting was eliminated. The facial esthetics, especially in lip tissues, was significantly improved. The lip protrusion and incompetence disappeared, and a normal nasolabial angle and labiomental fold morphology were achieved (Figure 5A‐C). A well‐aligned dentition with good interdigitation, proximal contacts, and ideal overjet and overbite was obtained (Figures 5D‐H and 6). The panoramic radiograph showed no significant root resorption or alveolar bone loss (Figure 7A). Cephalometric radiograph and tracing superimposition showed that both the dentoalveolar relationship and facial profile esthetics were greatly improved (Figures 7B and 8). Photographs taken 4 years after treatment indicated that the treatment stabilities in dental and facial tissues were both maintained (Figure 9). The mandibular movement was normal with no temporomandibular joint symptoms.

## 4 DISCUSSION

Treatment of Class II division 1 malocclusion includes growth modification, orthodontic treatment, and orthognathic surgery.^2^ In the present case, orthodontic treatment was a reasonable alternative for an adult patient without significant skeletal discrepancies. Nonextraction and extraction are both modalities in orthodontic treatment, and in the present case, nonextraction was performed for the following reasons: First, the facial profile convexity was acceptable with excellent soft‐tissue chin position, so the upper incisors should be retracted moderately to avoid flattening of midface.^5^ Second, the lower lip was in a more posterior position with lingual collapse of lower incisors, so lower incisors should be positioned anteriorly to restore the lost lower lip support.^13^ Thus, the upper incisor retraction should be restricted with its location based on the position of lower incisors. Third, the measurement of H angle was high but not far beyond the receivable range, so it could be improved by moderate upper lip retraction with nonextraction.^13^ Fourth, the Class II molar relationship was slight, which could be corrected by moving lower dentition anteriorly without extraction. Finally, arch length was manageable. Space in upper arch was available due to the diastemata in anterior segment and bilateral compression of upper arch. Crowding in lower arch was mild, which could be solved with lower incisors being moved anteriorly. During the orthodontic treatment, both upper and lower arches were aligned and leveled. Occlusal interruption was eliminated by using resin raisers. The upper and lower arches were improved in shape to coordinate with each other. The above factors all contributed to adequate retraction of upper incisors and establishment of normal overjet and overbite. However, some unfavorable factors such as excessive labial inclination of lower incisors and lingual inclination of upper incisors, which might lead to crowding relapse or mandibular dysfunction, need to be focused after treatment. Lower third molars need to be extracted, and some interproximal enamel reduction of lower incisors needs to be performed.

Optimal facial esthetics is an important goal as well as ideal dental occlusion in orthodontics.^1^, ^2^ Consideration of facial soft tissues is critical as well as skeletal and dental tissues in orthodontic diagnosis and treatment planning.^2^ In the present case, the patient showed much concern about her incompetent lip as well as the protrusive teeth. Therefore, abnormalities in soft tissues, including lip protrusion, lip incompetence, acute nasolabial angle, and deep labiomental fold, must be paid attention to as much as dental problems. It has been reported that perioral soft‐tissue profile, especially the lip form, could be improved by incisor retraction after orthodontic treatment.^3^, ^4^ However, soft tissues do not always respond favorably to changes in dental tissues for individual patients.^8^ The correlation between lip change and incisor retraction could be influenced by individual soft‐tissue traits and oral habits.^7^, ^8^, ^9^ It was found that upper lip thickness and strain played an important role in the prediction of soft‐tissue changes resulting from upper incisor retraction after orthodontic treatment.^8^ In patients with excessive lip strain or thin lip, the correlation between lip change and upper incisor retraction was significant, while in patients with insufficient lip strain or thick lip, the correlation was insignificant.^8^ Moreover, individual oral habits such as lip biting or sucking were sometimes the cause of abnormalities in both lip and dental tissues, and the opposite force from these abnormal muscular activities could counteract the effect of orthodontic treatment.^9^ Therefore, it was important to evaluate characteristics of soft tissues in determining treatment plan.^5^, ^7^ In the present case, soft‐tissue analysis showed insufficient strain of upper lip before treatment, indicating that the upper lip protrusion could hardly be changed solely by upper incisor retraction. Meanwhile, the patient had a habit of lower lip biting; thus, orthodontic treatment could not be enough to change soft‐tissue profile. In such case, conventional orthodontic treatment needed to be assisted by lip myofunctional training. Active myofunctional training could promote morphological improvement in soft tissues, which further facilitated orthodontic treatment to achieve optimal esthetics.^9^, ^10^, ^11^, ^12^

Finally, long‐term stability after orthodontic treatment needs to be considered. Perioral musculature dysfunction, typically insufficient lip strength, was found in patients with Class II division 1 malocclusion.^14^, ^15^ And this could lead to imbalance of strength between the inside and outside of dental arches.^16^ Therefore, myofunctional training is necessary to facilitate the long‐term dentoalveolar stability by improving lip function and establishing equilibrium within oral environment.^17^, ^18^, ^19^ In the present case, the patient was instructed to perform lip myofunctional training all through the treatment. Optimal facial esthetics was immediately achieved after 18 months of active treatment, and dentoalveolar stability was maintained at the 4‐year follow‐up.

## 5 CONCLUSION

It is critical to evaluate characteristics of soft tissues as well as skeletal and dental tissues in orthodontic treatment planning. Orthodontic treatment assisted by lip myofunctional training is an effective option to achieve optimal facial esthetics and long‐term dentoalveolar stability in adult patients with Class II division 1 malocclusion.

## CONFLICT OF INTEREST

None declared.

## AUTHOR CONTRIBUTIONS

JH: performed the orthodontic treatment in this case and wrote the manuscript; XM: performed the operation related to operative dentistry in this case.

## Data Availability

Data and materials are presented in the main paper.
